# Study protocol: evaluation of ‘JenMe’, a commercially-delivered weight management program for adolescents: a randomised controlled trial

**DOI:** 10.1186/s12889-015-1923-y

**Published:** 2015-06-19

**Authors:** Aimee L Dordevic, Maxine P Bonham, Robert S Ware, Leah Brennan, Helen Truby

**Affiliations:** Department of Nutrition & Dietetics, Monash University, Level 1, 264 Ferntree Gully Rd, Notting Hill, VIC 3168 Australia; UQ Child Health Research Centre, School of Medicine, The University of Queensland, QLD, 4006 Australia; School of Population Health, The University of Queensland, QLD, 4006 Australia; School of Psychology, Australian Catholic University, VIC, 3065 Australia

**Keywords:** Adolescent, Commercial weight management, Obesity

## Abstract

**Background:**

Early lifestyle intervention with overweight and obese adolescents could help to avoid serious health events in early adulthood, ultimately alleviating some of the strain on the public health system due to obesity-related morbidity. Commercial weight loss programs have wide reach into the community setting, and have demonstrated success in long term weight management in adults, beyond that of current public health care. Commercial weight-management programs have not been evaluated as a method of delivery for overweight and obese adolescents. This study aims to evaluate the efficacy of a new adolescent weight management program in a commercial environment.

**Methods:**

One hundred and forty adolescents, 13 to 17 years old, will be randomised to either a weight management program intervention or a wait-listed group for 12 weeks. The commercial program will consist of a combined dietary and lifestyle approach targeting improved health behaviours for weight-loss or weight-stability. Participants will be overweight or obese (above the 85^th^ percentile for BMI) and without existing co-morbidities. Outcome measures will be assessed at baseline and after 12 weeks. Primary outcome measures will be changes in BMI Z-score and waist-height ratio. Secondary outcome measures will include changes in behaviour, physical activity and psychosocial wellbeing. Intervention participants will be followed up at 6 months following completion of the initial program. Ethics approval has been granted from the Monash University Human Research Ethics Committee (CF11/3687–2011001940).

**Discussion:**

This independent evaluation of a weight management program for adolescents, delivered in a commercial setting, will provide initial evidence for the effectiveness of such programs; which may offer adolescents an avenue of weight-management with ongoing support prior to the development of obesity related co-morbidities.

**Trial registration:**

The protocol for this study is registered with the International Clinical Trials Registry ISRCTN13602313.

## Background

Australian prevalence of overweight and obesity in childhood and adolescence increased from 20.9 % in 1995 to 24.7 % in 2008 [1]. While the trend has started to stabilise, a significant proportion (25.7 % in 2012) of Australian children and adolescents aged 5 to 17 years, remain overweight or obese [1]. Within sub-populations the prevalence can be even higher, for example 31 % of children living in lower socioeconomic status (SES) areas, including rural areas are overweight or obese compared to 18 % of those living in the highest SES areas [2].

Overweight and obese adolescents are more likely to become overweight and obese adults than healthy weight adolescents [3]. Additionally, obesity-related cardiovascular disease (CVD) risk markers present in adolescence are predictive of adult, obesity related morbidity [4]. A study in Denmark observed that every 1 unit increase in body mass index (BMI) Z-score from age 7 to 13 years in boys and 10 to 13 years in girls, significantly increases the risk of a cardiovascular event in adulthood [5]. A 2001 Australian report suggested that interventions that result in as little as 5 kg weight loss in all overweight and obese individuals could lower the total prevalence of overweight and obesity by 15 % [6]. Thus, adolescent weight management is a significant, modifiable risk factor for the development of cardiovascular morbidity that could lead to premature mortality in adult life [7–9].

There have been two Cochrane reviews of childhood obesity management. The 2009 Cochrane Review considered interventions for treating obesity in children. It included a total of six weight-loss dietary intervention studies (*n* = 350 participants across age ranges) demonstrating overall beneficial effects of weight-loss on adiposity and BMI Z-score [10]. Twelve physical activity intervention studies were also included, which generally reported no significant change in BMI Z-score or adiposity [10]. Meta-analyses of lifestyle interventions demonstrated a favourable effect on BMI Z-score at 6 months [mean difference (MD) = -0.06 (95 % CI -0.12,–0.01)] in children aged less than 12 years, and in those over 12 years of age [MD-0.14 (95 % CI-0.17,–0.12)]. Parental involvement was noted to be an ingredient for successful intervention. However, the report highlighted the scarcity of studies in the adolescent age range.

The more recent 2011 Cochrane Review focused on interventions for preventing obesity in children and used BMI Z-score as a key outcome measure. The Review demonstrated the very different effects of weight management programs in children compared to adults, with data being abstracted and grouped by age rather than by intervention type. Most new studies included in 2011 were obesity prevention studies in children, the majority of which took place in schools. In the adolescent age group (13 to 18 years), analysis of ten studies demonstrated an overall BMI change of -0.09 kg/m^2^ (95 % CI-0.20, 0.03) [11]. However a high level of heterogeneity between studies was observed potentially due to inconsistent levels adiposity across studies as different cut-offs and anthropometric measures were applied. Further, the authors stressed an increasing awareness of the need for weight loss maintenance, and recommended that future studies monitor the effectiveness of programs in the longer-term [11].

A retrospective study that examined the long term effects of a weight management program for individuals (aged 1 to 18 years), delivered by physicians and dietitians in a primary care setting, demonstrated significant reductions in BMI Z-score [MD-0.16 (95 % CI not reported)], and participants subsequently showed improvements in several CVD risk factors such as LDL-cholesterol (*p* = 0.02), 2 h plasma glucose (*p* = 0.004) and peak insulin levels (*p* = 0.04) after a 2 year period [12]. Those who attended sessions more frequently exhibited better results. Improved blood pressure has also been observed in children and adolescents (aged 7 to 15 years) 2 years following an intensive 3-month dietary intervention with a strong follow-up program, that reduced BMI Z-score by-0.16 (95 % CI-0.22,–0.09) at 3 months [13].

Whilst programs delivered within primary care settings are modestly successful, publicly funded health systems in Australia tend to only accommodate individuals with existing obesity-related comorbidities. There are few treatment options for overweight and obese adolescents without complications. Early behavioural intervention targeted at reducing weight with longer-term maintenance could help reduce the numbers of young patients presenting to the public health system with serious weight-related health events. Evidence suggests obese young people who successfully lose weight do not have obvious complications arising from their obesity, such as insulin resistance [14].

Randomised controlled trials have demonstrated the superiority of commercially delivered programs for weight management in adults, when compared with usual care through the health service system [15–17]. Commercial weight loss programs are successful in initiating and maintaining long term weight loss in adults [18] and may also be a successful setting for weight management adolescents. Collaboration between commercial organisations and primary care facilities may be an efficacious strategy to deliver weight management programs on a large scale [19]. Commercial weight management programs can offer ongoing, long term support that cannot be provided by health professionals in primary care and has a wide reach including rural areas and they have the ability to be accessible to individuals within the population that are unable to access primary medical services for weight management [20]. Despite evidence for the benefits from an intensive multifactorial approach to weight-management in adolescents [10, 11, 21], no study to date has examined commercially available and delivered weight management programs specifically tailored to the needs of adolescents.

Jenny Craig Weightloss Centres Pty Ltd, has developed a new 12-week adolescent weight management program, ‘JenMe’. The program involves weekly face to face sessions with a Jenny Craig-trained consultant that includes dietary and behavioural education as well as monitoring of the progress of the adolescents. Menus and foods are provided initially, with support for the adolescent to eventually develop skills in their own menu planning. On completion of the 12 week program on-going support is available that can be tailored to the individual’s needs. The program has not yet been evaluated for efficacy, an essential first step in supporting the claim of an evidence-based approach to weight management in young people. This study aims to address this gap.

We propose to evaluate a commercially delivered adolescent weight management program ‘JenMe’; for efficacy. We know that lifestyle interventions for children are effective in the short term but there are few studies that examine the long term prognosis of short term weight loss and the ability of adolescents to maintain that weight loss over time. Data from the 6-month diet and lifestyle ‘Eat Smart’ intervention for Australian adolescents compared favourably with previously published data (BMI Z-score MD-0.12 (SD 0.10) at 3 months) [10], but early follow up data have already began to show a plateau in weight loss followed by some weight re-gain. Hence, the current study proposes to follow intervention participant weight management for 6 months after completion of the 12 week program.

This study will be the first to assess an adolescent weight management program that is conducted in a commercial environment. Its findings will inform the literature in the field of adolescent weight-loss and management, and has the potential to unveil an effective solution to adolescents seeking weight management in a community setting.

## Methods

### Study design and participants

**Fig. 1:**
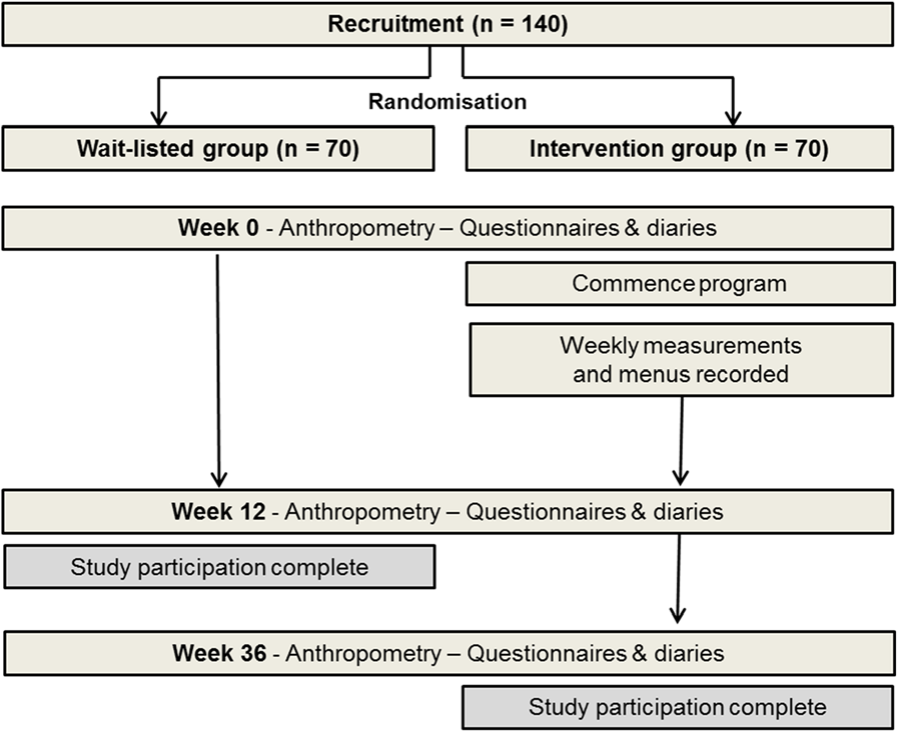
Study flow design. One hundred and forty adolescents (13 to 17 years, BMI Z-score ≥ 1.282) will be recruited and randomised 1:1 to either the intervention or wait-listed control group. The intervention group will receive a 12 week commercial weight management program. The wait-listed control group will be offered the program after the control period (12 weeks)

The current study is a multicentre, parallel-group randomised controlled trial. Adolescent boys and girls aged 13 to 17 years who apply to join the commercial weight management program will be screened for eligibility to participate. Individuals who do not meet the health screen requirement, a standard practice for enrolment to Jenny Craig weight management programs, will not be eligible for inclusion in the study. Exclusion criteria of the program include being aged less than 13 years or greater than 17 years; having diagnosed diabetes; being pregnant; having given birth to a child during adolescence; having a medical condition or using medications related to weight co-morbidities (except asthma). Individuals who have a BMI Z-score of ≥ 1.282, indicating that they are above the 85^th^ percentile for BMI for their age and gender, will be invited to participate in the study. Participants will be randomised into either the treatment group or the wait-listed control group. The treatment group will enter the weight management program immediately, and the control group will be wait-listed for 12 weeks. Figure 1 shows the study flow. Behavioural questionnaires and diaries will be completed during the week prior to the first consultation where anthropometric characteristics of participants will be recorded. The same questionnaires and diaries will be repeated during week 12 (the final week of the program for participants in the interventions group), and participants will again have anthropometric characteristics recorded at the end of week 12. Primary outcome measures will include changes in BMI Z-score and waist to height ratio. Secondary outcome measures will include changes in physical activity levels, behaviour and psychosocial wellbeing. Participants will have the option to volunteer to have further cardio-metabolic risk factors and body composition examined. All experimental procedures are being conducted in accordance with the Declaration of Helsinki and were formally approved by the Monash University Human Research Ethics Committee (CF11/3687–2011001940).

### Sample size calculations

For the purpose of sample size estimation, the primary outcome of this study was difference in BMI Z-score at 12 weeks. Our own pilot data, and data from the largest RCT in obese adolescents yet published (209 children aged 8-16 with BMI > 95^th^ percentile; Savoye 2007) suggest the standard deviation in BMI Z-score in a non-passive weight loss intervention will be approximately 0.27. Assuming a 2-sided 0.05 significance level, a sample size of 52 participants per group would provide 80 % power to detect a clinically important treatment difference of 0.15 BMI Z-score. To account for a 25 % drop-out, we plan to enrol 70 participants in each group.

### Recruitment and randomisation

The study will be advertised within the weight management centres in Victoria, Western Australia, New South Wales and the Australian Capital Territory as well as on the company website. Potentially eligible clients will be assessed in the weight management centres by consultants, guided by dietitians and/or a general practitioner for study eligibility. If participants who meet eligibility criteria are interested in participating in the study, they will be shown an information DVD. All participants will provide written consent that will be obtained by consultants in the weight loss centres from both the adolescent and their parent or guardian. Consenting adolescents will then be recruited into the study via telephone by a researcher.

Allocation to treatment group (intervention or wait-listed control) will occur using computer-generated random numbers. Group allocation will occur off-site by a statistician with no day-to-day study involvement in the trial. Individuals will be allocated 1:1 to the intervention group or wait-listed. Randomisation of the study participants will be stratified by the weight-management centre from which they are enrolled. Within each centre, randomisation will occur in blocks of either 6 or 8. Block size will be randomly selected and unknown to anyone except the study statistician (RSW). Treatment allocation will be stored in an online database, accessible only by the study statistician, and will remain unknown until revealed to the study co-ordinator on the day each participant is allocated to a treatment group. Due to the nature of the intervention masking participants to group allocation is not possible.

### Intervention: the JenMe program

A 12 week evidence-based lifestyle intervention program was developed by the team of Accredited Practising Dietitians at Jenny Craig Australia and was reviewed for content by paediatric psychologists specialising in behaviour change. The program will be delivered face to face to clients in the Jenny Craig Centres by consultants on a weekly basis for the duration of the program. Consultants in the centres have been trained by Jenny Craig and are not otherwise required to be trained health professionals. Specialised program materials will be used with participants and their parents or guardians. The participants will present to the centre to meet with a consultant weekly for 13 consecutive weeks (Weeks 0 to 12). Each week, a new element of the program (Table 1) will be delivered at each consultation to the adolescent, who will be encouraged to modify small behaviours each week. The order of topic deliverance will be directed by the adolescent and tailored to their needs. The parent or guardian will be involved in part of the consultation to ensure participation in the goal setting process. The program also encompasses a dietary plan that has been modelled on the dietary guidelines as outlined by the National Health and Medical Research Council’s (NHMRC, 2013) Australian Dietary Guidelines [22].

**Table 1:** Topics covered over 12 weeks within the JenMe program

Eat Well		Move more	Live life
▪ Decoding drinks		▪ How to move more by tracking how you move less	▪ Body image
▪ Eating out for success		▪ Overcoming your activity barriers	▪ Learning from lapses
▪ Jenny at school & friends’		▪ Setting SMART goals	▪ Rewards & buddies
▪ Meals with the family and fast & healthy after school snacks		▪ Time for a physical activity challenge!	▪ Saying No
▪ Non-hungry eating & learning to eat with awareness			
**Independent menu planning**			
Getting started on your own	1. Your own menu guide	2. Working out grocery serves of recipes	3. Working out grocery serves of packaged foods from labels

Initially the participant will follow the menu that includes pre-packaged food provided by the commercial program, combined with their own grocery items. Throughout the program the adolescent, with the support of their consultant and parent or guardian will learn how to plan their own menus using all of their own foods, and either during or following the 12 week program, they will eventually transition onto their own menu planning (Table 1). In accordance with usual practice at Jenny Craig, once the 12 week ‘JenMe’ program is complete, adolescents have the option to receive ongoing, individually-tailored support, depending on the need for further weight-loss or weight-maintenance up to the age of 18 years of age as part of their Jenny Craig membership. Also, as with any commercial program, participants are free to withdraw from the program at any stage, for any reason.

### Wait-listed control

The wait-listed control group will receive standard healthy eating guidelines, the ‘Healthy Eating for Children’ booklet developed by the National Health and Medical Research Council based on the Australian Dietary Guidelines [22], and advised to maintain their current lifestyle habits for the control period. Wait-listed control participants will be offered the commercial weight management program at the end of the control period (12 weeks).

All study participants, from both the intervention and control groups will receive a free adolescent membership with Jenny Craig, which is valid until they are 18 years of age as well as a discount on all Jenny Craig food provisions for 12 months.

### Outcomes

#### Anthropometry

All participants will have their baseline weight, height, waist, and body composition (using bioelectric impedance analysis (BIA); BODYSTAT Quad Scan 4000, BODYSTAT Ltd., Douglas, Isle of Man, UK) measured by trained Jenny Craig consulting staff using standard operating procedures. BMI Z-score will be calculated by the lambda-mu-sigma (LMS) method using Centre for Disease Control reference data (http://www.cdc.gov/growthcharts/percentile_data_files.htm) which takes into account age and gender. Participants will be required to self-assess their pubertal status using the Tanner stages of puberty scale [23, 24]. The intervention group will have weekly weight measurements, height measured every 4 weeks and waist and body composition assessed by BIA measured at 0 and 12 weeks. Wait-listed control participants will have all measurements taken at 0 and 12 weeks only.

#### Dietary analysis

Intervention and wait-listed control participants will be required to complete a 4-day food diary, both in the week prior to commencement of the intervention or wait-listed period and again at 12 weeks. Dietary analysis of energy and nutrient intake will be performed using Foodworks 7 (Xyris Software Pty Ltd., QLD, Australia). Foodworks will be supplemented with food composition data of the pre-packaged foods supplied by the commercial weight-management company for assessment of intervention participants’ week 12 energy and micronutrient intake.

#### Physical activity

In order to monitor physical activity levels, the adolescents in both groups will be required to complete the Adolescent Physical Activity Recall Questionnaire (APARQ), which has been validated in an Australian population [25]; and complete a 4-day activity diary (Children’s Nutrition Centre, University of Queensland, Australia). A pedometer will be worn over the 4- day period and number of steps will be recorded in the activity diary. This will be performed both in the week prior to commencement of the intervention or wait-listed period and repeated at 12 weeks.

#### Participant attitudes to eating, self-image and quality of life

Questionnaires will be completed by both groups both in the week prior to commencement in the study and again at week 12 of the intervention or wait-listed period. The questionnaires will be used to assess the impact of the program on psychosocial factors and also the relationship between baseline psychosocial factors and primary outcomes. General and body specific self-esteem will be assessed via the Rosenberg 5-item self-esteem scale questionnaire [26], and the Mendelson & White 24-item body-esteem questionnaire [27].

The Eating Attitudes Test (EAT)-26 [28] will be used to measure symptoms and concerns characteristic of eating disorders. The items on this scale will be summed to form three subscales; Dieting, Bulimia and Food Preoccupation and Oral Control.

The Strengths and Difficulties Questionnaire (SDQ) adolescent version report will be used to measure adolescent behaviours, emotions and relationships [29]. The items on this scale will be summed to form five subscales; Emotional Symptoms, Conduct Problems, Hyperactivity, Peer Problems, and Pro-social Behaviour.

The Impact of Weight on Quality of Life-KIDS (IWQOL-Kids) will be used to measure obesity specific health related quality of life [30]. The items on this scale will be summed to form four subscales; Physical Comfort, Body Esteem, Social Life, Family Relations.

The 12-item multidimensional perceived social support questionnaire will also be completed to assess three sources of perceived support: family, friends, and significant other [31].

These measures have been validated for use with children and adolescents [26–28, 30, 32–34].

#### Parental involvement

Parents and guardians will be required to complete the Strengths and Difficulties Questionnaire (SDQ) parent version report, a measure of adolescent behaviours, emotions and relationships (Subscales; Emotional Symptoms, Conduct Problems, Hyperactivity, Peer Problems, and Prosocial Behaviour) [29] and a demographic questionnaire prior to their adolescent’s commencement in the study and the SDQ will be repeated during week 12 of the intervention or wait-listed period.

#### Metabolic markers

Participants from both groups will be able to volunteer for further metabolic testing. A subset of participants will opt-in to provide a fasting blood sample at 0 and 12 weeks for the assessment of metabolic markers. Blood will be collected in EDTA containing blood tubes (McFarlane Medical & Scientific, NSW, Australia) and immediately centrifuged at 2000 x g for 15 min at 4 °C. Samples will be stored at-80 °C until analysis.

Glucose, cholesterol and triacylglycerol (TAG) concentrations in plasma will be measured on a Roche Cobas Integra 400 plus auto-analyser (Roche, Lavel, Quebec, Canada) by enzymatic colorimetric methods using commercially available kits (CHOL2, TRIGL and Glucose HK Gen 3) as per the manufacturer’s instructions (Roche, Lavel, Quebec, Canada).

Plasma insulin will be determined using the Human Insulin Specific RIA kit HI–14 K (Merck Millipore, Billerica, MA, USA) according to manufacturer’s instructions and read on a Gamma Counter.

#### Body composition using dual energy x-ray absorptiometry (DXA)

Participants from both groups will be able to volunteer for further body composition assessment. A subset of individuals will opt-in to participate in a whole body DXA scan for body composition at 0, 12 and 36 weeks (intervention only). Participants will attend the laboratory and have their height and weight measured, using standard operating procedures, to determine the correct amount of radiation required for the whole body scan. Whole body composition will be estimated with a GE LUNAR iDEXA Narrow-Angle Dual Energy X-Ray Densitometer with SmartFAN™ (GE Medical, Software Lunar DPX enCORE 2012 version 14.0, Madison, WI).

### Follow-up

All outcome measures will be assessed in the intervention participants at a follow-up at 36 weeks after commencement in the program (6 months after program completion), in order to track longer-term weight management. It will not be possible to follow-up the wait-listed participants as a control group as they will have the option to commence the program at end of their 12-week wait-listed period.

### Statistical analysis plan

Summary descriptive statistics for demographic and clinical data at baseline will be reported by allocated study treatment. Continuous data will be summarised descriptively using either mean and standard deviation, or median and inter-quartile range, depending on the distribution of the variable of interest. Categorical data will be presented as frequencies and percentages. Comparisons between the treatment groups will be conducted to assess the degree to which comparability of randomisation was achieved.

The primary study outcomes are between-group differences in age and gender adjusted BMI Z-score and waist-to-height ratio at 12 weeks. The mean difference between treatment groups will be calculated using linear regression with treatment group entered as the main effect and covariable adjustment for baseline outcome. The corresponding 95 % Wald confidence interval and p-value will be reported. For secondary outcomes, the effect estimate relating to a variable with a binary outcome will be presented as an odds ratio, which will be calculated using a logistic regression model. The effect estimate relating to a variable with a continuous outcome will be presented as a mean difference, which will be calculated using a linear regression model. The effect estimate relating to a variable with a count outcome will be presented as an incident rate ratio, which will be calculated using a Poisson regression model. For all models considering outcomes measured at 12 weeks, the baseline outcome will be included as a covariable. For all models the corresponding 95 % Wald confidence interval and p-value will be reported.

The initial approach to all analyses will be based on the ‘intention to-treat’ (ITT) principle, where all evaluable data is analysed in the treatment group according to which the participant was allocated. Additionally, because of the potential for differential between-group attrition due to the nature of recruitment, all analyses will be investigated to see whether results are sensitive to possible between-group imbalances in key demographic, social, or clinical characteristics. Characteristics of participants for whom follow-up data is available will be compared between groups, and variables significantly different at P < 0.05 will be identified. Multivariable regression models will be run with treatment group as the main effect and covariables including the value of the outcome at baseline and characteristics identified as differing between treatment groups at follow-up. All sensitivity analyses will be clearly labelled., Statistical significance will be defined as alpha = 0.05. All tests conducted will be two-tailed.

Outcomes measured at six months will be analysed using mixed-effects regression models with a random intercept for each participant. For continuous outcomes Gaussian family and identity link function, for binary outcomes a binomial family and logit link function, and for count outcomes a Poisson family and log link function will be used. All models will include time period (baseline/12 weeks/36 weeks) as a main effect, and other covariables will be included as appropriate.

### Ethics and dissemination

It has previously been demonstrated that children randomised to wait-listed or only given written information (control groups) gain weight [35]. Allocating overweight and obese children to be wait-listed for 12 weeks raises ethical concern as weight gain in children that are not treated could be disadvantaged, particularly with children that have identified their need for weight-loss assistance in a community setting. In the current study, as participants will not have weight-related co-morbidities, ethical permission has been approved to delay treatment in a wait-listed control group for 12 weeks after which they will have the opportunity to undertake the commercially available weight management program. Participants have the right to withdraw from the study at any time, for any reason. If a wait-listed control participant chooses to commence the program immediately, it will be made available to them, but they will not participate in the study. Ethical approval for the study has been granted by Monash University Human Research Ethics Committee (approval no: CF11/3687–2011001940).

## Discussion

Tailored weight management strategies are needed to reduce the growing prevalence of adolescent overweight and obesity, and consequent burden of disease in young people. However, interventions must be delivered with care to avoid the promotion of harmful teenage dieting and take precautions to ‘do no harm’. Body image is an highly ranked concern among Australian adolescents [36]. Concerns over body image have been linked to engagement in dieting behaviours that can lead to disordered eating [37]. A USA study showed that of 2287 surveyed adolescents 61 % of girls and 28 % of boys had engaged in unhealthy weight-loss behaviours [38]. Furthermore, the incidence of these behaviours has been observed to increase with age [39]. The JenMe adolescent program is designed to engage and educate adolescents using evidence-based principles for healthy eating and activity behaviours, rather than promote dieting with unhealthy weight-management outcomes. The validated questionnaires that are being used to assess the safety and efficacy of the program are designed to evaluate whether the program can nurture positive outcomes rather than facilitate harmful behaviours.

Commercial programs are currently available within the community and have demonstrated to be successful in an adult population [15, 17]. Such programs have the resources to provide ongoing, long term support that is not always provided by health professionals in primary care due to restraints on time and patient load [20]. This study is designed to determine whether a multi-faceted, commercial weight management program, delivered in a community setting will be a useful resource for overweight and obese adolescents.

This study will observe changes in body measures including BMI Z-score and waist to height ratio, and outcomes such as quality of life, in order to thoroughly assess the efficacy and safety of the program.
